# Determining the Contextual Factors in a Decision‐Making Framework for a Rugby League Ball Carrier: A Rapid Review and Delphi Study

**DOI:** 10.1002/ejsc.12271

**Published:** 2025-02-15

**Authors:** James Bletsoe, Sarah Whitehead, Jamie Poolton, Kevin Till

**Affiliations:** ^1^ Carnegie Applied Rugby Research Centre Carnegie School of Sport Leeds Beckett University Leeds UK; ^2^ Leeds Rhinos RLFC Leeds UK

**Keywords:** attacking play, coaching, decision making, performance analysis, rugby league

## Abstract

Using a two‐phase approach in the form of a rapid literature review and Delphi consensus, this study aimed to reach consensus on the terms, definitions and potential options to develop a framework that captures the contextual factors that can affect a rugby league ball carrier’s decision‐making, whilst also determining the perceived importance of these contextual factors. Forty terms, their definitions and potential options were extracted from the rapid review. In a two‐round Delphi survey, experts rated their level of agreement with each term, definition and potential options on a five‐point Likert scale. Consensus was defined by ≥ 80% agreement (with ≤ 10% in disagreement). The experts then rated the level of importance to a ball carrier’s decision‐making of each of the terms on a seven‐point Likert scale. Eighteen experts participated in round one and 15 participated in round two (response rate 83%). Five additional terms were suggested by the experts and reached consensus in the second round of the Delphi survey. In total, consensus was reached on 45 terms, their definitions and potential options, which were grouped into five themes (match context, offensive context, defensive context, offensive ball carrier skill and attacking outcomes). Seventeen of the 45 terms were perceived to be *important* or *very important*. Nine of these factors were associated with offensive context and eight factors were associated with defensive context. The framework can be used by coaches, performance analysts and researchers to better understand player in‐game decisions and to support the design of training interventions.

**Summary**

- This is the first study to reach consensus on contextual factors that could affect a rugby league player’s decision‐making.
- 45 contextual factors, their definitions and potential options reached consensus forming a framework to better understand a rugby league ball carrier’s decision‐making.
- Nine offensive and eight defensive contextual factors were deemed to be either important or very important, indicating the significance of both facets of the game on a ball carrier’s decision‐making.
- This framework can be used in both practice and research to better understand in game decision‐making.

## 1 Introduction

In team invasion sports, such as rugby league, it is widely agreed that an athlete's capability to make decisions under pressure plays a significant role in performance (Berry, Abernethy, and Côté 2008; Kinrade, Jackson, and Ashford 2015; McGuckian, Cole, and Pepping 2018). Although extensive research on decision‐making has been conducted in laboratory‐based settings (Ashford, Abraham, and Poolton 2021a; Inns et al. 2023), it is understood that task relevant information or contextual factors play an important role within the decision‐making process (Williams, Ward, and Chapman 2003; McRobert et al. 2011; Farrow et al. 2018). Levi and Jackson (2018) (p19) defined contextual factors as the ‘*circumstances before and during a match that influence decision making*’. In rugby league, there are a wide range of in‐match contextual factors that have previously been cited by players, such as defensive line speed, tackle count or speed of the last play the ball, which have been suggested to have an impact on an individual player's decision‐making process (Johnston and Morrison 2016). However, no consensus has been reached on what all of these contextual factors might be and how these can form a framework for practitioners and researchers.

It has been argued that a lack of research exploring contextual factors in ecologically valid game contexts is limiting the practical implications for coaches that can be drawn from the existing literature (Collins, Collins, and Carson 2022). More recently, research has started to address this in soccer and rugby union, respectfully (Levi and Jackson 2018; Collins, Collins, and Carson 2022; Ashford, Abraham, and Poolton 2021b). Through player interviews, it was established that there are a wide range of contextual factors that influence, to a greater or lesser degree, a player's decision‐making in team invasion sports (Levi and Jackson 2018; Collins, Collins, and Carson 2022; Ashford, Abraham, and Poolton 2021b). Research into the contextual factors at play in a sport has wide ranging applications for coaches and analysts in terms of decision‐making evaluation and development through training practices.

Gabbett and Abernethy (2012) established that decision‐making played an important role in the scoring of tries in the Australasian National Rugby League with nearly 50% of tries being scored from a draw and pass situation, which involves attacking players making decisions based on contextual factors (e.g., attacker *v* defender ratio and positioning of supporting runner). Further to this, Connor, Crowther, and Sinclair (2018) showed that elite players focus on different cues (or contextual factors) and movement patterns in comparison to novice players, highlighting the importance of identifying game and skill level specific contextual factors. However, this study focused on only two evasive manoeuvres and visual behaviour within the game and did not include specific contextual factors. This work is supported by Johnston and Morrison (2016) who explored the use of cues in the decision‐making process in professional and semi‐professional rugby league players. Their results indicate that there was a difference in the use of cues, with professional players demonstrating greater cue discrimination and the tendency to process cues in a different way to semi‐professional players. For example, higher skilled players in this study reported using a smaller number of cues and the ability to identify and provide meaning to the most effective cues. Furthermore, this study was one of the first to identify contextual factors that player's use as a part of the decision‐making process in rugby league.

The importance of decision‐making in differentiating playing levels in evasive manoeuvres (Pearce et al. 2020) and core skill (Pearce et al. 2019) has been explored in rugby league. Pearce and colleagues (2019, 2020) compared pass, tackle and attacking evasive manoeuvre decision‐making through a battery of field‐based tests between under 18, under 20 and state level players. It is suggested that more skilled individuals can perceive more global or big picture factors, such as the width of the defensive line or the attacker versus defender ratio; whereas less skilled players rely on more discrete local information, such as the body positioning of an opponent (Johnston and Morrison 2016). The type of contextual factors used may be determined by the amount of time available to process them (Ashford, Abraham, and Poolton 2021b). If a player has more time, they may be able use the more global cues to make a decision; whereas if they have less time, they may tend to use more discrete cues (Basevitch et al. 2019). These studies suggest that higher level players have greater success in effectively and efficiently making decisions. Moreover, the research implies that the importance of a specific contextual factor in the decision‐making process is dependent on the skill level or the time available to the player making the decision. However, although these studies do identify some contextual factors, currently, no research has established a consensus on what the factors at play in rugby league are or has the research established the potential importance of these factors.

Research within rugby league has determined what individual or team match actions relate to success or level of play (Gabbett 2014; Kempton, Sirotic, and Coutts 2017; Parmar et al. 2017; Woods, Sinclair, and Robertson 2017; Woods et al. 2018; Whitehead et al. 2020). Parmar et al. (2018) identified the components of ‘amount of possession’ (e.g., metres gained) and ‘speed of play’ (e.g., line breaks) as indicators of successful match outcomes. In another example, Woods, Sinclair, and Robertson (2017) highlighted five significant match actions (i.e., try assists, all attacking run metres, line breaks, number of dummy half runs and offloads) in successful National Rugby League performances. Of note, all of these are attacking match actions whilst a team is in possession of the ball. Bletsoe et al. (2024) also established the importance of attacking match actions along the rugby league player pathway in England. Players from the academy and scholarship teams that went on to play in the European Super League demonstrated better attacking match actions than players that did not. As attacking match actions have been established as significant differentiators in match play, it is pertinent to understand the role contextual factors play in the decision‐making process of the player in possession of the ball (i.e., the ‘ball carrier’).

In rugby league, it has been established that a ball carrier’s actions are important to success (Parmar et al., 2018), that decision‐making plays an important role in the execution of match actions (Scott et al. 2021) and that contextual factors play an important role in the decision‐making process (Johnston and Morrison 2016). To the authors’ knowledge, there has been little research attempting to capture the contextual factors and their importance, that may affect decision‐making of the ball carrier and none that has reached a consensus on these factors.

Some interesting contextual factors that have been suggested could play a role in a ball carrier’s decision‐making are their playing position and the position on the field of play (middle of the field compared to the edge of the field). As Dixon et al. (2023) discuss, players are presented with different challenges based on their playing position, role within the team based on this position and potential contact and collision scenarios. Players have highlighted the different roles they play when playing as a middle forward compared to an adjustable (including half back). When playing as an adjustable, their role was to create more space for edge players (Johnston and Morrison 2016; Dixon et al. 2023); whereas when playing as a middle forward, their role involved more carrying into contact (Dixon et al. 2023). Cupples and O'Connor (2011) also established that players in different positions have specific roles within the team and that they have an impact on a player’s decision‐making. Decision‐making was highlighted as important for all positions but more so for adjustable in attack (Cupples and O'Connor 2011), which is logical as they touch the ball more than any other position (Sirotic et al. 2011). The position on the field was also an important consideration for players as their prepared for contact. When carrying the ball in middle of the field, players felt that they were able to anticipate and brace for contact more based on what they could see in front of them, whereas when they carried the ball on the edge of the field, they were confronted with more ‘blind side’ contacts, preventing them from fully preparing for contact (Dixon et al. 2023). This anticipation of contact could have a direct impact on the decision‐making process of a player.

Morgan, Mouchet, and Thomas (2020) highlighted that some coaches look to implement a specific criteria for evaluating in‐game decision‐making creating a more objective process. The creation of a framework to capture the in‐game contextual factors could assist in this process and ensure consistency when coding match events for coaches and analysts (Mackay et al. 2023). By reaching a consensus on these contextual factors, coaches, performance analysts and researchers can speak in a common language when working on in‐game decision‐making of the ball carrier and defensive systems. Within the proposed framework, the contextual factors (‘terms’) would have a potential range of ‘options’; for example, for the ‘term’ *defensive line speed,* it is important to establish what the different types or ‘options’ of *defensive line speed* are, such as *slow, moderate* or *fast*. Finally, it is important that all ‘terms’ and ‘options’ within a framework that can be used for analysis are clearly defined to ensure reliable coding (Williams, 2012). Each of these factors can then be grouped into themes to form the framework (Hendricks et al. 2020). Once the framework is developed, the perceived importance of contextual factors can be ascertained. Knowledge generated regarding the potential significance of each of the factors within the decision‐making process may validate or challenge a coach’s viewpoint and provide a reference point for coaches and analysts to prioritise contextual factors in match evaluation and training practices.

Based on the above, the aims of this study were to

- reach consensus on the terms, definitions and potential options that describe the contextual factors that can affect a rugby league ball carrier’s decision‐making and the outcome of the decision;
- organise the contextual factors and the outcomes into a decision‐making framework for a rugby league ball carrier;
- determine the perceived level of importance of these contextual factors to establish which are the most important in the ball carrier’s decision‐making process.

## 2 Methods

To achieve the three study aims, a two‐phase approach was utilised; Phase‐one: a rapid review of literature and Phase‐two: a two‐round expert Delphi consensus method. The methodology was developed from previous framework consensus studies in rugby league (Hopkinson et al. 2021), netball (Mackay et al. 2023) and rugby union (Hendricks et al. 2020). Institutional ethics approval was obtained (ref: 118621).

### 2.1 Phase‐One

In phase‐one, a rapid review of literature (Smela et al. 2023) was conducted to extract terms and definitions previously used within research on rugby league and rugby union to describe contextual factors and match actions in attacking play. The electronic database SPORTDiscus was searched using the terms ‘rugby’ OR ‘rugby league’ OR ‘rugby union’, in combination with ‘video analysis’ OR ‘performance analysis’ OR ‘decision making’ OR ‘framework’ OR ‘attack’ OR ‘offense’ OR ‘performance indicators’ OR ‘match action’ from inception until September 2023. Terms (i.e., an element of match play that could either affect a ball carrier's decision‐making or an outcome of a particular ball carry), definitions (i.e., the operational definition of that term) and options (i.e., the potential specific scenarios from that element of match play) were extracted from research publications. The authors reviewed the terms used within these articles and made any relevant adjustments. For example, rugby union terminology was converted into rugby league terminology (e.g., ‘Break down’ or ‘Ruck’ into ‘Play the Ball’). Other terms, definitions and options were added from the STATS Perform Manual (STATS Perform 2021). STATS Perform are the official data provider to the two major rugby league professional leagues, the European Super League (The RFL 2023) and National Rugby League (STATS Perform 2018). The authors, who have extensive research and practice experience in rugby league talent development and high‐performance environments, individually proposed terms that were seen to be missing from the initial terms, definitions and options. These terms had to be agreed upon by all members of the authorship team to be included (Mackay et al. 2023). Terms and definitions were categorised into five themes: three context specific (i.e., ‘match context’, ‘offensive context’ and ‘defensive context’) and two outcome specific (i.e., ‘ball carrier offensive skill’ and ‘attacking outcome’). These initial themes were determined by the authors (Mackay et al. 2023) based upon themes that emerged from the rapid literature review. These themes and the terms that went into each theme had to be agreed upon by all members of the authorship team (Mackay et al. 2023).

### 2.2 Phase‐Two; Delphi

Phase‐two consisted of a two‐round Delphi consensus method (Hasson, Keeney, and McKenna 2000; McMillan, King, and Tully 2016) to establish consensus on the terms, their definitions, their options and overall framework assembled in Phase‐one. The Delphi was conducted between November and December 2023 using an online software Qualtrics (Qualtrics, Provo, United States of America) and is reported following the ACCORD (Accurate Consensus Reporting Document) checklist (Gattrell et al. 2024). The experts were informed that, for the purposes of this research, the term ‘ball carrier’ referred to any player in possession of the ball during a play.

#### 2.2.1 Experts

Using a purposeful sampling technique, potential experts were identified by the authors based upon the inclusion criteria below and were invited to participate via email (*n* = 45) (Palinkas et al. 2013). Consideration was given to inviting an equal number of experts from each criteria (Mackay et al. 2023), as all roles were seen as important by the authors to understand a ball carrier’s decision‐making. These potential experts were encouraged to forward the email to anyone else whom they felt might fit the criteria. To be included in the study, experts were required to meet one or more of the following criteria: (a) currently (or within the last 2 years) a coach or performance analyst in a high‐performance rugby league setting (i.e., performance pathway, professional and international) with a minimum of 3 years’ experience; (b) published research regarding decision‐making or technical/tactical performance analysis in rugby league or (c) currently a player in a professional rugby league setting with a minimum of 5 years’ experience. All experts were over 18 years of age and were based from either the United Kingdom, Australia or New Zealand, as this is where the two elite leagues are based (European Super League and Australasian National Rugby League). Consideration was given to recruiting a diverse panel (e.g., sex/gender and ethnic origin), but consideration was not given to the socio‐economic status. The authors aimed to ensure representation from different experts, countries and playing standards.

A panel of 18 experts was established. To achieve reliable results, a Delphi panel should contain > 10 experts (Vergouw et al. 2011). There were 18 respondents to the first round of the survey. Fifteen of these experts then also completed the second round of the survey (83% response rate). Of the experts that completed both rounds of the survey, all were male and were from either the United Kingdom or Australia; five were coaches, with an average experience of 19.8 ± 4.4 years and three coaching at the international level, one in top level professional club rugby (National Rugby League or European Super League) and one on a player pathway. Two experts were current players, both having played internationally, with an average experience of 13.5 ± 3.5 years. Six experts were performance analysts with an average experience of 9.0 ± 3.2 years; whereby four had worked at the international level and two in top level professional club rugby. Two experts had published relevant research in rugby league. Written informed consent was obtained from all experts.

#### 2.2.2 Delphi Round One

In round one, each expert was asked to rate their level of agreement for each of the terms, definition and options within the framework established in Phase‐one on a five‐point agreement Likert scale (Mackay et al. 2023) (1—strongly disagree, 2—somewhat disagree, 3—neither agree nor disagree, 4—somewhat agree and 5—strongly agree). Experts were provided with an opportunity to make any comments and/or suggestions about the terms, their definitions, their options and changes to the overall themes. Consensus was reached for each term, definition and options if ≥ 80% agreement (i.e., ‘strongly agree’ and ‘somewhat agree’), with ≤ 10% disagreement (i.e., ‘strongly disagree’ and ‘somewhat disagree’), was achieved between the expert panel (Hasson, Keeney, and McKenna 2000). The experts were given 2 weeks to respond. The terms, definitions and options that did not reach consensus in this round were reworded based on the suggestions made by experts in preparation for round two. The experts were given the opportunity to make suggestions for any new terms, definitions or options to be included within the framework. These suggestions had to be approved by all members of the authorship team before being included in the second round. The experts were also given an opportunity to make any additions to the themes.

#### 2.2.3 Delphi Round Two

In round two, a second round of agreement ratings were attained for the themes, their definitions and options that did not reach consensus in round one (*n* = 5), including the additions suggested by experts in round one (*n* = 5). The median (interquartile range [IQR]) for each of the variables that did not reach consensus were reported alongside the terms, definitions and options, allowing for reflection on the ratings from round one. Consensus was reached if ≥ 80% agreement, with ≤ 10% disagreement, was achieved between the expert panel (Hasson, Keeney, and McKenna 2000).

**FIGURE 1:**
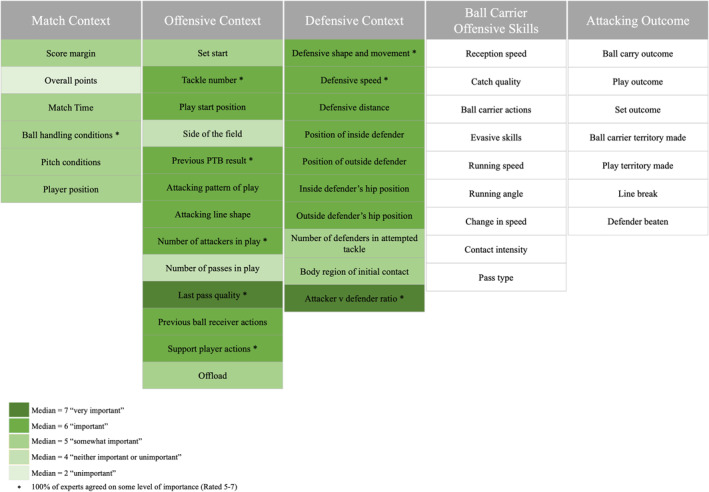
The overall framework of the themes and terms included in the consensus.

Experts were also asked to rate the perceived level of importance to a ball carrier’s decision‐making of each of the terms in the three context‐based themes (*match context, offensive context* and *defensive context*) (see Figure 1) on a seven‐point Likert scale (Costa 2005) (1—very unimportant, 2—unimportant, 3—somewhat unimportant, 4—neither important nor unimportant, 5—somewhat important, 6—important and 7—very important). This was conducted as a seven‐point Likert scale to allow respondents to choose between more clearly opposed options based on their opinions and increase the sensitivity of results between the contextual factors rather than just looking to reach a level of agreement (Finstad 2010). No options for comments were included in the section. The experts were given 2 weeks to respond.

Responses were downloaded into a Microsoft Excel file for analysis. The level of agreement for each theme after the second round for each of the terms, their definitions and options is reported as median (IQR). The perceived level of importance is reported as median (IQR) and similar to Heyward et al. (2022), who utilised a five‐point scale; Likert scale ratings were combined (i.e., unimportant: 1–3; neither important nor unimportant: 4 and important: 5–7) for percentage responses.

## 3 Results

Forty initial terms, definitions and options that were used to create the initial framework for ball carrier’s decision‐making, which were obtained from 13 research publications (Johnston and Morrison 2016; Pearce et al. 2020; Hendricks et al. 2013, 2020; Hopkinson et al. 2021; Laird and Lorimer 2004; Sayers and Washington‐King 2005; Gabbett, Kelly, and Pezet 2008; Wheeler and Sayers 2009; Wheeler, Askew, and Sayers 2010; Wheeler, Wiseman, and Lyons 2011; Austin, Gabbett, and Jenkins 2011; den Hollander et al. 2016) (*n* = 23), STATS perform (2021) (*n* = 10) and authors discussion (*n* = 7, 100% of terms suggested by the authors were agreed for inclusion).

In round one of the Delphi, 35 terms and their definitions and options reached consensus and five new contextual factors were proposed. The five factors that did not reach consensus were reworded and re‐rated in round two alongside the five additional terms proposed by the expert panel. Following round two, all terms, their definitions and options reached agreement. There were no comments on changing the overall themes and the location of the factors in the framework (Figure 1). The final terms, definitions and options are detailed in Tables 1, 2, 3, 4, 5. The median (IQR) rating of agreement was 5.0 (0.0) for the match context theme (Table 1), the offensive context theme (Table 2), the defensive context theme (Table 3), the offensive ball carrier skill theme (Table 4) and the attacking outcome theme (Table 5).

**TABLE 1:** Match context terms, definitions and options.

Term and options	Definition
Score margin	The points margin between the teams at the start of the carry.
Overall points^b^	The number of points, from both teams combined, that have been scored in the match up to that point.
Match time	The time on the official match clock at the start of the carry.
10 min increments	
Ball handling conditions	The conditions for the ball carrier at the start of the carry.
Wet (rain or snow)	
Humid	
Dry	
Pitch conditions^b^	The pitch conditions for the ball carrier at the time of the carry.
Firm	
Slippery	
Heavy	
Artificial	
Player position	The playing position of the player carrying the ball.
Full back	
Wing	
Centre	
Half	
Middle forward	
Hooker	
Edge back row	

*Note:* No ^a^ and ^b^ indicates the term, definition and options reached agreement after round one of the consensus.

^a^Term, definition and options reached agreement after round two of the consensus.

^b^ Term, definition and options added after round one, and agreed after round two, of the consensus.

**TABLE 2:** Offensive context terms, definitions and options.

Term and options	Definition
Set start	The method that the current set begins.
Kick off	
Scrum	
Tap	
Play the ball	
Kick return	
Opposition error	
Set restart	
Drop out	
Tap 20	
Tackle number	The phase of play (or tackle count) in which the ball carry takes place.
Play start position^a^	The position on the field at the start of the current phase (tackle count).
(One of the following):	
0–10 m	0–10 m from the team in possession’s try line.
11–20 m	11–20 m from the team in possession’s try line.
21–30 m	21–30 m from the team in possession’s try line.
31–40 m	31–40 m from the team in possession’s try line.
41–50 m	41–50 m from the team in possession’s try line.
51–60 m	51–60 m from the team in possession’s try line.
61–70 m	61–70 m from the team in possession’s try line.
71–80 m	71–80 m from the team in possession’s try line.
81–90 m	81–90 m from the team in possession’s try line.
91–100 m	91–100 m from the team in possession’s try line.
(and one of the following):	
Wide left	Outside the left‐hand tap line.
Left	Between the left‐hand scrum line and left‐hand tap line.
Left middle	Between the left‐hand post and left‐hand scrum line.
Middle	Between the posts.
Right middle	Between the right‐hand post and right‐hand scrum line.
Right	Between the right‐hand scrum line and right‐hand tap line.
Wide right	Outside the right‐hand tap line.
Side of the field^b^	The side of the field the carry took place on in relation to where the last play the ball was.
Open side	The wider side of the field from where the last play the ball took place. If the play the ball was exactly in the middle of the field, this will be the side with the most attacking players.
Short side	The shorter side of the field from where the last play the ball took place. If the play the ball was exactly in the middle of the field, this will be the side with the least attacking players.
Previous PTB result^a^	The result of the previous tackle events post contact phase (which is either by grounding of the tackler or called by the referee) to the end of the play the ball.
Attacking ruck win	Play the ball was 3 s or less **and/or** 2 markers were not set to defend **and/or** the defensive line was not set for more than a second.
Attacking ruck neutral	Play the ball was between 4 and 5 s **and** 2 markers were set to defend **and** the defensive line was set for 1–3 s.
Attacking ruck lost	Play the ball was over 6 s **and** 2 markers were set to defend **and** the defensive line was set for over 3 s.
Attacking pattern of play^a^	The width of attack with respect to the distribution of the ball along the attacking line.
Dummy half run	When the attacking ball carrier received possession of the ball directly from the play the ball, commonly called ‘scoot’.
Ruck carry	When the attacking ball carrier received possession of the ball through a short pass from the play the ball. The ball carrier runs back to the direction of the ruck. Tries to get in behind the markers.
Hit up	When the attacking ball carrier received possession of the ball through a pass immediately from the play the ball and runs into the defensive line at the first defender (commonly called A defender) or wider.
First receiver	When the attacking ball carrier received possession of the ball through a pass from the dummy half but does not go into contact.
Plus one attack	When the attacking ball carrier received possession of the ball through a pass from the first receiver.
Plus two attack	When the attacking ball carrier received possession of the ball through a pass from the second receiver.
Wide attack	When the attacking ball carrier received possession of the ball through a pass from outside a third receiver and is outside the scrum line opposite to where the play the ball was.
Counter‐attack	When the attacking ball carrier received possession of the ball through an opposition turn over and play continued.
Kick return	When the attacking ball carrier received possession of the ball through a kick from the opposition and play continued.
Restart return	When the attacking ball carrier received possession of the ball through a kick off or dropout from the opposition and play continued.
Second phase attack	When the attacking ball carrier received possession of the ball through a continuation of the phase. An example of receiving the ball from a second phase is when an attacking ball runner makes contact with the defense and then offloads the ball to a team member in support who then continues play.
Attacking line shape	The formation of the attacking team at the start of the play.
(Can be more than one):	
Flat	Attacking players were positioned in a horizontal line.
Deep	Attacking players were positioned in a diagonal line.
Wide	Attacking line is spread across the width of the field.
Narrow	Attacking line is bunched, close to the breakdown.
Number of attackers in play	The number of attackers actively involved in that play, including all ball receivers and support runners that attract the attention of a defender.
Number of passes in play	The number of passes up to the ball carrier receiving the ball in that play.
Last pass quality	The quality of the pass from the previous ball receiver
Good	The passed ball was received by the ball receiver between their chest and hip height. Ball receiver did not need to make any postural or line of motion adjustments to receive possession of the passed ball.
Moderate	The passed ball was received by the ball receiver between their chest and head height or between hip and knee height. Ball receiver had to make minor adjustments to posture or line of motion to receive possession of the passed ball (e.g., slowing running speed to catch the passed ball).
Poor	The passed ball was received by the ball receiver through extended reaching above head height or below knee height. The ball receiver had to make considerable adjustments to posture or line of motion to receive possession of the passed ball (e.g., diving or running backwards to catch the passed ball).
Previous ball receiver actions	The action of the last player to touch the ball.
Receive and pass	Previous ball player received the ball and passed it without engaging a defender.
Draw and pass	Previous ball player received the ball and engaged a defender before passing the ball.
Dropped ball	Previous ball player dropped the ball.
Offload	Previous ball player offloaded the ball in contact.
Kick	Previous ball player kicked the ball.
Support player actions^a^	The movement of the ball carrier’s teammates off the ball.
Genuine support	Support player ran a line with the clear intent of receiving the ball that attracted the attention of a defender who shows a clear physical response (e.g., defender holds on line runner or puts themselves in a position to tackle the ball carrier and/or support runner).
Passive support	Support player ran a line without the clear intent of receiving the ball and did not illicit a clear physical response from a defender (e.g., defender does not slow their movement for a line runner)
No support	There were no support players that ran a genuine line that attracted the attention of a defender.
Offload	A ball carrier in the current phase attempted to pass the ball in the process of being tackled.
No offload	There was no offload in that play.
Complete offload	There was an offload that went straight to the hands of a teammate on that play.
Offload to ground	There was an offload that went to ground on that play before being collected by a teammate.

*Note:* No ^a^ and ^b^ indicates the term, definition and options reached agreement after round one of the consensus.

^a^ Term, definition and options reached agreement after round two of the consensus.

^b^ Term, definition and options added after round 1, and agreed after round 2, of the consensus.

**TABLE 3:** Defensive context terms, definitions and options.

Term and options	Definition
Defensive shape and movement	The configuration and movement pattern of defenders.
Up and in	Defenders approached the attacking line in a straight‐line formation followed by the outer players (players furthest way from the ball) advancing ahead of the line towards the ball.
Up and out	Defenders approached the attacking line in a straight‐line formation followed by inner players (players closest to the ball) following the movement of the ball towards the touchline.
Push/rush	The defenders approached the attacking line at a fast speed and are in a straight and direct line.
Lateral shift	Initial movement of the defenders is towards the touch line without challenging attacking line/attacker.
Advancing runner	A defender shot rapidly from the defensive line ahead of the other defenders towards the ball carrier.
Edge jam	A defender/defenders shot rapidly from the defensive line ahead of the other defenders outside of the ball carrier.
Arrow head	Defenders approached the attacking line in a triangle shape formation; that is, one defender is followed by other defenders besides and behind them on each side.
Static line	Defenders were in a straight line with no movement towards the attacking line/attacker.
Defensive speed	Speed of the defence in response to the attacking line, when the first ball carrier in the phase receives possession of the ball.
Slow	Stationary or walking (no locomotor movement). Slow forwards, backwards or sideward movement. One foot in contact with ground at all times and no arm drive.
Moderate	Jogging or a slow run with low knee lifts and little arm drive.
Fast	Running with high knees and rapid arm movement or sprinting at ball reception.
Defensive distance	Distance of the defence in relation to the ball carrier when they receive the ball.
Close	Attacker received the ball within 2 m of a defender.
Moderate	Attacker received the ball between 2 and 5 m from a defender.
Distant	Attacker received the ball more than 5 m from a defender.
Position of inside defender	The playing position of the defender on the inside of the ball carrier as they receive the ball.
Full back	
Wing	
Centre	
Half	
Middle forward	
Hooker	
Edge back row	
Position of outside defender	The playing position of the defender on the outside of the ball carrier as they receive the ball.
Full back	
Wing	
Centre	
Half	
Middle forward	
Hooker	
Edge back row	
Inside defender’s hip position^a^	The hip position of the inside defender.
Turned in	The inside defender’s hips turned inwards, towards where the last play the ball was.
Square	The inside defender’s hips remained straight on, parallel with the field.
Turned out	The inside defender’s hips turned outwards, away from where the last play the ball was.
Outside defender's hip position^a^	The hip position of the outside defender.
Turned in	The outside defender’s hips turned inwards, towards where the last play the ball was.
Square	The outside defender’s hips remained straight on, parallel with the field.
Turned out	The outside defender’s hips turned outwards, away from where the last play the ball was.
Number of defenders in attempted tackle	The number of defenders committed to attempt to tackle the ball carrier.
Body region of initial contact	Where the tackler struck the ball carrier on initial contact.
Head and neck	The tackler initially struck the area above the shoulder with any connection with the head/neck.
Shoulder	The tackler initially struck any area from the ball carrier’s arm‐pit level to the shoulder level including the arm.
Torso	The tackler initially struck the area above the ball carrier’s hips to arm pit.
Legs	The tackler initially struck the ball carrier’s legs below the hips.
Ball	The tackler struck the ball initially.
Attacker *v* defender ratio^b^	The ratio of the number of players in the attacking line compared to the defensive line at the start of the play on that side of the play the ball. This could be open side or blind side.
Man on man	Same number of attackers and defenders.
One man overlap	One more attacker in the attacking line compared to the defensive line.
Two man overlap	Two more attackers in the attacking line compared to the defensive line.
Multiple overlap	Over two more attackers in the attacking line compared to the defensive line.
One man underlap	One more defender in the defensive line compared to the attacking line.
Two man underlap	Two more defenders in the defensive line compared to the attacking line.
Multiple underlap	Over two more defenders in the defensive line compared to the attacking line.

*Note:* No ^a^ and ^b^ indicates the term, definition and options reached agreement after round one of the consensus.

^a^ Term, definition and options added after round 1, and agreed after round 2, of the consensus.

^b^ Term, definition and options reached agreement after round two of the consensus.

**TABLE 4:** Ball carrier offensive skill terms, definitions and options.

Term and options	Definition
Reception speed	The speed at which the ball carrier was running as they received the ball.
Standing	No locomotor activity.
Walking	Locomotor moment with no flight phase and minimal arm swing.
Jogging	Locomotion with a flight phase and minimal arm swing.
Cruising (or striding)	Similar to jogging with a more active arm swing.
Sprinting	Maximal locomotor activity.
Catch quality	The quality of the ball carrier’s catch irrespective of the previous pass quality.
Good	The ball receiver had their hands and fingers up, palms out and took the pass early. This takes into consideration the quality of the pass.
Poor	The ball receiver did not either have their hands and fingers up, palms out or did not take the pass early (caught the ball in their body). This takes into consideration the quality of the pass.
Ball carrier actions	The actions of the attacking ball carrier.
Carry into contact	The ball carrier took the ball into an attempted tackler without any attempted evasive skills.
Evasive carry	The ball carrier attempted to evade a defender/defenders.
Receive and pass	The ball carrier received the ball and attempted to pass it without engaging a defender.
Draw and pass	The ball carrier received the ball and engaged a defender before attempting to pass the ball.
Offload	The ball carrier took the ball into contact and attempted to offload the ball.
Kick	The ball carrier kicked the ball.
Evasive skills	Movements or actions that coerce an opponent into a movement pattern that is then exploited by the ball carrier.
(can be more than one):	
2 *v* 1	Move the defender away from the support player, deliver a timed pass to the support player.
Dummy pass deception	A feigned pass
Dummy kick deception	A feigned kick
Side‐step	Agility manoeuvre initiated from the outside leg.
Crossover‐step	Agility manoeuvre initiated from the inside leg.
Skip	Change of tempo (slow to fast). Permits maintenance of balance to affect rapid change of direction.
No fend	The ball carrier provided no fend.
Moderate fend	The ball carrier provided a light to moderate fend (e.g., swat or slap technique).
Strong fend	The ball carrier provided a strong fend (e.g., push technique).
Run angle left, pass left	Angle run to the left and pass ball to left.
Run angle left, pass right	Angle run to the left and pass ball to the right.
Run angle right, pass left	Angle run to the right and pass ball to the left.
Run angle right, pass right	Angle run to the right and pass ball to the right.
Behind flick pass	The ball carrier passes the ball with a flick of the wrist behind their torso.
Running speed	The speed of the ball carrier once they have received the ball.
Slow	Stationary or walking (no visible foot movement).
Moderate	Jogging (low knee lift).
Fast	Running or sprinting (high knee lift).
Running angle	Running line or direction of the ball carrier towards defence.
Straight	Ball carrier ran straight at the defence.
Arcing	Ball carrier ran in a wavy line at defence.
Lateral	Ball carrier ran laterally, from touchline to touchline.
Diagonal	Ball carrier ran in a straight angled line at defence.
Straighten	Ball carrier changed running angle from any angle to vertical (relative to touchline).
Turn	Ball carrier changed running angle from any angle to an angle that us not vertical.
Change in speed	The ball carrier displays a visible change in speed prior to contact.
Acceleration	Ball carrier increased running speed.
Deceleration	Ball carrier decreased running speed.
No change	Ball carrier did not change running speed.
Contact intensity	The actions of the attacking ball carrier when in contact with the defense.
Good	The ball carrier displayed a good body height. Technical indicators also included a strong leg drive with the ball carrier not submitting to the tackle and then advancing the ball beyond the tackle line.
Moderate	The ball carrier displayed an average body height. Technical indicators also include an initial leg drive from the ball carrier but then submitting to the tackle of the defense or the ball carrier being tackles equal to the tackle line.
Poor	The ball carrier displayed a high body height. Technical indicators also included a poor leg drive, submissive in contact or being driven behind the tackle‐line.
Pass type	If the ball carrier attempted to make a pass, the type of pass they attempted.
Short lateral pass	Standard pass to receiver, within a 5 m radius.
Long lateral pass	Standard pass to receiver, further than a 5 m radius.
Miss pass	Ball was transferred past the closest player to the ball carrier further away.
Flat pass	Ball was transferred horizontally, so that the receiver runs onto the ball when catching it.
Inside ball	Ball was passed to the receiver running on the inside channel of the passer.
Lob pass	High looping pass.
Pop pass	Short pass initiated from the wrists, rather than the arms, to the receiver in the immediate proximity of the ball carrier.
Quick hands	Ball was received and passed to the receiver in one rapid movement.
Switch pass	Ball was transferred in the opposite direction of the previous pass.
Pass to ground	Ball touched the ground from a pass before the receiver retrieved it.

*Note:* No ^a^ and ^b^ indicates the term, definition and options reached agreement after round one of the consensus.

^a^Term, definition and options reached agreement after round two of the consensus.

^b^Term, definition and options added after round 1, and agreed after round 2, of the consensus.

**TABLE 5:** Attacking outcome terms, definitions and options.

Term and options	Definition
Ball carry outcome	The result at the end of the ball carry when the ball carrier has released the ball.
Try scored	
Error	
Kick	
Penalty conceded	
Penalty won	
Attacking ruck win	Play the ball was 3 s or less **and/or** 2 markers were not set to defend **and/or** the defensive line was not set for more than a second.
Attacking ruck neutral	Play the ball was between 4 and 5 s **and** 2 markers were set to defend **and** the defensive line was set for 1–3 s.
Attacking ruck lost	Play the ball was over 6 s **and** 2 markers were set to defend **and** the defensive line was set for over 3 s.
Pass	
Offload	
Play outcome	The result of the play.
Try scored	
Drop goal	
Error	
Kick	
Penalty conceded	
Penalty won	
Attacking ruck win	Play the ball was 3 s or less **and/or** 2 markers were not set to defend **and/or** the defensive line was not set for more than a second.
Attacking ruck neutral	Play the ball was between 4 and 5 s **and** 2 markers were set to defend **and** the defensive line was set for 1–3 s.
Attacking ruck lost	Play the ball was over 6 s **and** 2 markers were set to defend **and** the defensive line was set for over 3 s.
Repeat set	
Handover	
Scrum won	
End of half	
Set outcome	The result of the set.
Try scored	
Drop goal	
Error	
Kick	
Penalty conceded	
Penalty won	
Repeat set	
Handover	
Scrum won	
End of half	
Ball carrier territory made	The number of metres gained over the advantage line by the ball carrier before releasing the ball.
< 0 m	
0–5 m	
5–10 m	
10–15 m	
15–20 m	
20–30 m	
30+ m	
Play territory made	The number of metres gained over the advantage line of that phase of play (tackle count).
< 0 m	
0–10 m	
10–15 m	
15–20 m	
20–30 m	
30+ m	
Line break	An attacking player breaks the defensive line in open play.
Ball carrier line break	The ball carrier broke the defensive line.
Phase line break	The ball carrier had a direct impact on a teammate making a line break on the same phase of play.
No line break	There was no line break on that phase of play.
Defender beaten	
Ball carrier defender beaten	The ball carrier performed an offensive manoeuvre or broke an attempted tackle that led to a defender missing a tackle.
Phase defender beaten	The ball carrier had a direct impact on a teammate breaking an attempted tackle that led to a defender missing a tackle.
No defender beaten	There were no defenders beaten on that phase of play.

*Note:* No ^a^ and ^b^ indicates the term, definition and options reached agreement after round one of the consensus.

a Term, definition and options reached agreement after round two of the consensus.

b Term, definition and options added after round 1, and agreed after round 2, of the consensus.

Table 6 displays the perceived importance of each of the contextual factors on a ball carrier’s decision‐making, ranked from most important to least important. Nine of the contextual factors were deemed to have some level of importance by 100% of the experts (rated 5–7) with two of these achieving a median of 7 (7%) (‘very important’) rating. Fifteen contextual factors had a median of 6 (52%) (‘important’); nine had a median of 5 (31%) (‘somewhat important’); two had a median of 4 (7%) (‘neither important nor unimportant’) and one contextual factor had a median of 2 (3%) (‘unimportant’).

**TABLE 6:** Perceived level of importance of contextual factors on a ball carrier’s decision‐making.

Term	Theme	Median (IQR)	Important (5–7)	Neither important nor unimportant (4)	Unimportant (1–3)
Attacker *v* defender ratio	Defensive	7.0 (1.5)	100%	0%	0%
Last pass quality	Offensive	7.0 (1.5)	100%	0%	0%
Defensive shape and movement	Defensive	6.0 (1.0)	100%	0%	0%
Number of attackers in play	Offensive	6.0 (1.0)	100%	0%	0%
Previous PTB result	Offensive	6.0 (1.0)	100%	0%	0%
Support player actions	Offensive	6.0 (1.0)	100%	0%	0%
Defensive speed	Defensive	6.0 (1.5)	100%	0%	0%
Tackle number	Offensive	6.0 (1.5)	100%	0%	0%
Previous ball receiver actions	Offensive	6.0 (1.0)	93%	7%	0%
Defensive distance	Defensive	6.0 (1.0)	93%	7%	0%
Position of outside defender	Defensive	6.0 (1.0)	93%	7%	0%
Attacking line shape	Offensive	6.0 (1.5)	93%	0%	7%
Outside Defender’s hip position	Defensive	6.0 (2.0)	93%	0%	7%
Position of inside defender	Defensive	6.0 (0.5)	87%	7%	7%
Attacking pattern of play	Offensive	6.0 (1.0)	87%	13%	0%
Play start position	Offensive	6.0 (1.0)	87%	7%	7%
Inside defender's hip position	Defensive	6.0 (1.0)	80%	7%	13%
Ball handling conditions	Match	5.0 (1.0)	100%	0%	0%
Number of defenders in attempted tackle	Defensive	5.0 (1.0)	80%	20%	0%
Pitch conditions	Match	5.0 (1.0)	80%	0%	20%
Set start	Offensive	5.0 (1.0)	67%	20%	13%
Match time	Match	5.0 (1.5)	67%	20%	13%
Score margin	Match	5.0 (2.0)	67%	7%	27%
Offload	Offensive	5.0 (2.5)	67%	33%	0%
Player position	Match	5.0 (1.5)	60%	27%	13%
Body region of initial contact	Defensive	5.0 (1.5)	53%	33%	13%
Number of passes in play	Offensive	4.0 (2.0)	47%	27%	27%
Side of the field	Offensive	4.0 (3.0)	40%	27%	33%
Overall points	Match	2.0 (3.0)	20%	13%	67%
Overall defensive context		6.0 (2.0)	88%	8%	4%
Overall offensive context		6.0 (1.0)	83%	10%	7%
Overall match context		5.0 (2.0)	66%	11%	23%

## 4 Discussion

There is a dearth of research into a player’s decision‐making process in rugby league (Pearce et al. 2020). Although some research has previously taken place exploring a player’s decision‐making (Johnston and Morrison 2016; Connor, Crowther, and Sinclair 2018), no consensus had been reached on the specific contextual factors that play a role in the decision‐making process or, equally as importantly, defined these factors. Using a two‐phase approach in the form of a rapid literature view and Delphi consensus, this study aimed to establish consensus on a framework of the contextual factors that could influence a rugby league ball carrier’s decision‐making, which includes the potential outcomes of that decision‐making process. Forty‐five terms, 29 of which were contextual factors and 16 of which were outcomes, reached consensus. The contextual factors were organised into three themes (6 in *match context*, 13 in *offensive context* and 10 in *defensive context*) and the 16 outcomes were organised into two themes (9 in *ball carrier offensive skills* and 7 in *attacking outcome*) (See Figure 1). Additionally, the study quantified the views of an expert panel (players, practitioners and researchers) on the perceived importance of those contextual factors in the ball carrier’s decision‐making process. Forty‐two terms (93%) were determined to have a level of importance (median ≥ 5), with the median of the 17 of the terms being rated important or very important. These results and framework provide coaches, analysts and researchers in rugby league decision‐making a standardised set of operational definitions, ensuring consistency (Williams, 2012) and facilitating research and player development in this area (Hendricks et al. 2020; Hopkinson et al. 2021).

One potential practical application of the framework is that it can be used by coaches and performance analysts in an applied setting to review player decision‐making, diagnose errors and provide feedback. As O'Connor and Larkin (2015) identified, coaches have a strong reliance on performance analysis in evaluating decision‐making and performance. This framework could be used by performance analysts in video analysis software to capture the specific contextual factors of interest and connect them to key performance indicators (outcome measures), which can be mapped over time. The framework is purposefully descriptive, so that coaches can use the framework as a tool to implement their own philosophy. One consideration for practitioners and researchers alike is to ensure consistency when using the framework by both using the definitions provided (Williams, 2012), ensuring familiarisation with the framework, test–retest before using the framework and conducting a form of reliability testing whilst applying the framework (O'Donoghue 2007). Furthermore, the framework could be used by coaches as a reference point for discussions with players in one‐to‐one feedback meetings (Morgan, Mouchet, and Thomas 2020).

As with previous consensus studies (Mackay et al. 2023; Hendricks et al. 2020), the terms and their definitions can be used to assist with various aspects of the sport’s research. These terms and definitions, whilst related to decision‐making in this context, can be applied to other areas of interest in the game and could also be used in other areas of research, such as performance, talent identification or potentially injury‐based research. However, a primary objective of this framework is to provide a reference point for further exploration into on field rugby league decision‐making. As an example, the framework could be used to assess whether players on different levels make different decisions based on the contextual factors they are presented with expanding on previous research (Johnston and Morrison 2016; Connor, Crowther, and Sinclair 2018; Pearce et al. 2020). It has been established that there are different physical (Till et al. 2015, Till, Scantlebury, and Jones 2017) and match action (Bletsoe et al. 2024) demands on different levels of the European Super League player pathway, but no research has established whether players on the different levels are faced with different contextual factors and the decisions that player’s make in response to these demands. As discussed by Bletsoe et al. (2024), players on the lower levels of the player pathway face different match action demands, raising questions as to whether players are being adequately prepared and developed for Super League. As these physical and match demands are different, it would be logical that the contextual factors players face could be different on different levels of the pathway. Exploring this would help towards building a more holistic picture of the similarities and differences on the player pathway, having wide ranging implications for talent identification and development. Further research could also look to explore if players involved in more positive match actions make different decisions based on specific contextual factors and how best to manipulate contextual factors to train decision‐making in a practice environment (Collins, Collins, and Carson 2022).

Forty‐two terms (93%) were determined to have some level of importance (median ≥ 5 and ≥ 50% of experts rated as at least ‘somewhat important’) with eight of the terms (*attacker v defender ratio, last pass quality, defensive shape and movement, number of attackers in play, previous PTB result,* *support player actions, defensive speed and tackle number*) being rated overall as important or very important and all experts rated as at least ‘somewhat important’. Nine other contextual factors (*previous ball receiver actions, defensive distance, position of outside defender, attacking line shape, outside defender*’*s hip position, position of inside defender, attacking pattern of play, play start position and inside defender*’*s hip positions*) were also deemed important or very important. Interestingly, of these 17 factors deemed important or very important, 9 (53%) of these terms were in the *offensive context* theme and 8 (47%) were in the *defensive context* theme, highlighting the perceived importance of what is happening on both sides of the ball to player decision‐making. This supports the work of Johnston and Morrison (2016) in rugby league and Ashford, Abraham, and Poolton (2021b) in rugby union which highlighted that a range of contextual factors created by both a player’s own team and by the opposition play an important part of the decision‐making process.

As Passos et al. (2008) discuss, team sports, such as rugby league, are a complex dynamic system whereby a player’s actions can have a causal effect on teammates and opposition actions, where a range of factors interact with each other. It is important to consider some of the interdependency of these factors from both teams and the nonlinear nature of team sports. Coaches are well placed to recognise interactions between offensive and defensive contextual factors and their potential contribution to decision‐making. As an example from this framework, the *previous PTB result* could have a direct impact on the *defensive speed*, which could have an impact on the *previous ball receiver*’*s actions*. This was also highlighted in the work of Johnston and Morrison (2016), who suggested that players do not use contextual factors in isolation but can identify associations between factors and act based upon them. As Scott et al. (2021) discuss, there is an ongoing regulation between information, perception and action as these range of factors constantly evolve and interact between multiple individuals. If this perception of contextual factors happens collectively, coordinated team behaviours and actions can occur (Scott et al. 2021). The interaction of contextual factors should be explored in future research as well as the player’s and team’s abilities to link the causal effects of these factors, which could have a significant effect on a player’s ability to process multiple factors and impact collective team behaviours.

Of note, none of the factors that were deemed as having a high level of importance were in the *match context* theme and the *match context* theme had the lowest overall rating of importance of the three contextual themes with *overall points* the only contextual factor that was deemed unimportant by the expert panel. The one anomaly being that *ball handling conditions* was deemed as having a level of importance by 100% of the experts but not deemed a high level of importance (Median = 5). The overall results for the *match context* theme perhaps imply that on each individual carry the overall game status is not as important as what is happening specifically on that play or the more micro level events, such as *last pass quality* or *defensive distance* (Collins, Collins, and Carson 2022). This slightly contradicts Levi and Jackson (2018) who found the score status to have been an important factor in decision‐making, but this was in soccer, where scoring is less frequent so potentially has a bigger impact.

Another interesting point of note is that within the factors deemed important, there are both global (bigger picture cues, such as *defensive shape and movement*) and discrete factors (a more localised stimuli, such as *inside defender hip position*) (Johnston and Morrison 2016). As previously mentioned, a player’s position could play an important role in which type of contextual factors are being utilised more by a player (Johnston and Morrison 2016; Dixon et al. 2023). As discussed, adjustables might be looking to create more space for edge players (Johnston and Morrison 2016; Dixon et al. 2023), which implies that they may be looking at more global factors, such as *defensive shape and attacker* versus *defender ratio,* when deciding on what attacking shape to use. However, as the play develops, they be using more discrete factors, such as *outside defender*’*s hip position,* when deciding which option to take within that attacking shape. Middle forwards may have a different role, which involves taking the ball into contact, making metres and getting attacking ruck wins. In this case, they may rely more heavily on discrete cues, such as the *inside defender*’*s hip position* or *defensive distance. Player position* of the ball carrier is included within the framework to help explore this further. One direction for future research could be to build on the work of Dixon et al. (2023) and Johnston and Morrison (2016) through questioning players, aiming to establish whether players in different positions differ in their use of discrete and global factors.

In rugby union, Ashford, Abraham, and Poolton (2021b) and Collins, Collins, and Carson (2022) also established the fact that players use a wide breadth of both global and discrete factors when making decisions. These global factors are likely an important contributor to player’s decision‐making process at an elite level, but as Basevitch et al. (2019) discuss, if players have less time, the game information used becomes more discrete. Two of the terms were deemed to be very important: *attacker versus defender ratio* and the *last pass quality. Attacker versus defender* ratio would be considered more of a global factor, where a player would need to assess both the attacking players in play as well as the number of defenders on that side of the field. Whereas, the *last pass quality* would be more of a discrete skill that determines the time and information available to the ball carrier to make a decision and, in turn, the potential options afforded to them. Again, this implies the importance of both global and discrete skills in the decision‐making process. As Ashford, Abraham, and Poolton (2021b) discuss, global factors (*attacker vs defender ratio*) may take more time to identify. Highly skilled players may be more able to integrate global information into the decision‐making process than less skilled players (Johnston and Morrison 2016). Given the perceived importance of *attacker versus defender ratio* and that the use of global information, such as this characterises expertise, training activities should be designed that regularly expose ball carriers to player overloads and underloads (Gabbett and Abernathy 2012).

Although this was the first study to create a framework of the contextual factors that could influence a rugby league ball carrier’s decision‐making, and so the first to determine the relative perceived importance of those factors, it is not without limitations. The study did include at least two of each of the types of experts identified in the criteria, including nine with international experience. However, the inclusion of more than two players could have been beneficial in obtaining more opinions from experts currently performing ball carries in a competitive environment. Future studies could look to include more players and validate the perceived importance of the contextual factors. Only the offensive side of the game was explored in this framework, future studies could look to develop frameworks for defensive and transition elements of the game.

The framework also only includes in‐game contextual factors and does not consider external elements, such as game plans or prior knowledge of the opponents and self. As Broadbent et al. (2019) discuss ‘contextual priors’, whereby players come into the game with pre‐determined knowledge of factors can play a role in the decision‐making process. Although there is little research specifically examining the common practice in sport of previewing opposition, gameplans or own team philosophy via video, some studies have highlighted the importance of ‘contextual priors’ to players in game decision‐making (Levi and Jackson 2018; Collins, Collins, and Carson 2022; Ashford, Abraham, and Poolton 2021b; McLoughlin et al. 2023). Rugby Union players in a study by Collins, Collins, and Carson (2022) highlighted that knowledge provided to them on the opposition players before the game played a role in their decision‐making process, especially when they have more time to process the factors presented to them. McLoughlin et al. (2023) also identified the pre match context (*coach tactics and instructions, match importance and opposition status)*, alongside in game contextual factors, as one of the key themes that affect decision‐making for Gaelic football players. Interestingly, these ‘contextual priors’ also develop throughout the game as players gain more knowledge of their opposition (Ashford, Abraham, and Poolton 2021b). It has also been established that ‘contextual priors’ of a player’s own ability, past experiences and previous decisions made through video analysis of their own performance can also play an important role in the decision‐making process (Groom and Cushion 2004; Reeves and Roberts 2013). Although the aim of the present study was to exclusively establish the in‐game contextual factors, it is acknowledged that ‘contextual priors’ would be a factor in the decision‐making process and this interaction should be explored further in future research.

## 5 Conclusion

Forty‐five terms, their definitions and potential options explaining the contextual factors that could affect a ball carrier's decision‐making and potential outcome measures reached consensus following a two‐round Delphi survey. These factors were categorised into five themes (three contextual: *match context, offensive context* and *defensive context* and two outcome: *ball carrier offensive skills attacking outcome*). Seventeen contextual factors were deemed to be important or very important to a ball carrier’s decision‐making. Nine of these factors were offensive and eight were defensive implying both what a player’s team and what the opposition are presenting both play an important role in the decision‐making process. The framework developed through this study can be used in an applied setting by coaches and performance analysts as part of assessing a player’s decision‐making when ball carrying based on the game specific, task relevant information they are presented with. The aim is that this framework remains objective, and practitioners can apply it to their own team philosophies and game plans and be integrated as part of a wider assessment process. The recommendation would be for users of the framework to conduct reliability testing to ensure consistency. These terms can also be used by researchers to provide standardisation across both decision‐making literature and coaching/performance analysis studies. The next stages could be: using the framework to explore whether different playing levels face different contextual factors and whether players make different decisions based on those factors; establish whether more successful players make different decisions based on different contextual factors; determine whether players in different positions differ in their use of global and discrete factors and explore the interactions between ‘contextual priors’ and in game contextual factors.

## Conflicts of Interest

The authors declare no conflicts of interest.
